# Emerging evidence for gene mutations driving both brain and gut dysfunction in autism spectrum disorder

**DOI:** 10.1038/s41380-020-0778-5

**Published:** 2020-05-27

**Authors:** Beate Niesler, Gudrun A. Rappold

**Affiliations:** 1 Institute of Human Genetics, Department of Human Molecular Genetics, Heidelberg University, 69120 Heidelberg, Germany; 2 Interdisciplinary Center for Neurosciences (IZN), Heidelberg University, 69120 Heidelberg, Germany

**Keywords:** Autism spectrum disorders, Genetics

**Functional gastrointestinal disorders that are common in autism may derive from gene mutations previously linked to behavioral symptoms in autism spectrum disorder (ASD)**.

There is increasing awareness that patients suffering from neurological disorders including neurodevelopmental and neuropsychiatric diseases often present with gastrointestinal (GI) dysfunction, perceived as pain, stress, or discomfort. To date, the genetic factors associated with neuronal dysfunction in the brain and central nervous systems (CNS) have not been considered relevant in shaping GI symptoms. This has, however, recently changed. GI dysfunction has been described in various neurodevelopmental and psychiatric disorders including autism spectrum disorder and animal models have provided the first insights into how gut function is affected in these disorders [1].

Changes in microbiota composition and the presence of a leaky gut caused by impaired epithelial barrier function have been shown to contribute to changes in CNS structure and function relevant to neurological phenotypes [2, 3]. Within the past decade, evidence has accumulated that suggests microbiota-mediated gut–brain axis impairment also contributes to the pathogenesis of ASD and other disorders [1, 2, 4]. How far the complex pathogenesis is influenced by the host’s genetics has been the focus of research groups worldwide.

Here, we wish to highlight the genetic predispositions to ASD using established transcriptional profiles of the brain and gut. Strikingly, more than 90% of the 62 highest-ranking autism risk genes in the SFARI database (https://gene.sfari.org/) are expressed in both brain and GI tissues according to the genotype-tissue GTEx database (https://gtexportal.org/), suggesting they mutually affect the brain and the gut. Moreover, all listed SFARI genes are expressed in murine neural crest cells and enteric neural crest-derived cells that give rise to both peripheral and enteric neurons and glia, and the fetal gut (unpublished data). The expression of so many genes associated with ASD in the GI tract, including the enteric nervous system (ENS) as well as brain regions from embryonic to adult stages, suggests that these proteins might play a fundamental role in the development of the peripheral and CNS.

ASD is a neurodevelopmental disorder with altered behavior as a core feature. Clinically, ASD is characterized by impaired social interaction and communication, limited interests and activities as well as repetitive behaviors, suggesting impaired neuronal function. Moreover, autism severity has been associated with the probability of having GI problems [5].

The pathophysiology of GI dysfunction in ASD is still poorly understood. This may be attributed to the large clinical heterogeneity, multiple underlying genetic causes, environmental factors, and dysbiosis that lead to different developmental manifestations; but awareness of GI complaints in ASD has increased. Assessing GI dysfunction is challenging since symptoms such as pain, discomfort, heartburn, or nausea are difficult to assess and interpret because of difficulties in communication and altered pain perception. Therefore, it is largely unknown whether GI symptoms occur due to GI dysfunction or whether they represent an epiphenomenon caused by altered behavior or side effects of psychotropic medication.

To address this issue, several groups have set up questionnaires for children with ASD and their parents to assess GI symptoms in a standardized way. Studies implementing these systematic evaluations confirmed that feeding problems, functional diarrhea, and constipation as well as gastro-esophageal reflux disease are much more common in children with ASD than in healthy individuals [6].

Mutations in several ASD-associated genes have already been reported in patients with upper and lower GI disorders including achalasia and slow-transit constipation. *CHD8* mutations, for example, have been reported in patients with ASD that often presented with constipation. This was one of the first examples of an unexpected association between disturbed brain function and altered ENS development [7]. *NOS1* mutations were identified in patients diagnosed with autism and achalasia, resulting into eating and drinking problems [8], and *FOXP1* mutations were found in patients with ASD symptoms, language impairment, anxiety, and various GI complaints. Pitt–Hopkins syndrome is caused by *TCF4* mutations and is characterized by moderate to severe intellectual disability and ASD-associated behavioral deficits; in addition to these symptoms, patients frequently suffer from constipation and gastro-esophageal reflux.

Some studies have now also investigated the GI tract in animal models of ASD. Animal studies previously focused on brain alterations and associated behavioral consequences, while the GI tract has received only limited attention. This has recently changed, and studies are now addressing this issue, thereby underlining the importance of ASD genes in GI phenotypes. Patterns of gut motility in the GI tract of humans are remarkably similar to those of the mouse, meaning findings from these model systems can be extrapolated to human phenotypes [9]. Consistent with the phenotype seen in patients, mice with *Tcf4* haploinsufficiency, for example, present with impaired upper GI transit and disturbed rectal motility, leading to constipation [10]. Achalasia and delayed GI transit caused by impaired function of the lower esophageal sphincter and decreased motility, leading to eating and drinking problems and constipation, was observed in *Foxp1*^+/−^ mice [11]. Moreover, *gain-of-function* mutations in the serotonin transporter gene *Slc6a4* led to ASD-like behavior and a GI phenotype characterized by ENS hypoplasia and slow GI transit in mice [12]. Recently, mutations in neuroligin-3 (*Nlgn3*) led to GI dysfunction characterized by colonic dilation and accelerated GI transit, suggesting imbalances in neuronal activity [13, 14].

The relevance of ASD genes in GI phenotypes was also corroborated by recent findings of intestinal dysmotility in zebrafish with mutant *shank3a* and *shank3b* [15]. Impaired enteric neurogenesis and intestinal motility was reported for *Chd8* disruptions in zebrafish, recapitulating the human phenotype of constipation [7]. All these studies are in line with the idea that impairment of CNS-relevant genes might also affect ENS development and impact GI structure and function. These observations also support our hypothesis that impaired GI function, such as achalasia, reflux, and slow-transit constipation are not only comorbidities or epiphenomena but also as much a part of the ASD phenotype as the behavioral symptoms. Therefore, these data indicate that genetic defects affecting CNS structure and function in ASD might also affect ENS/GI function, which may explain the high prevalence of GI symptoms seen in ASD.

Abnormal development or disturbed structure and function of the ENS is associated with the development of neuropathic GI motility disorders such as achalasia, pseudo-obstruction, or functional constipation [16]. GI motility is orchestrated by a complex regulation and coordinated action of the ENS, smooth muscles cells, and interstitial cells of Cajal (ICCs). Developmental defects caused by gene variants may affect specific cell types or disturb the function of these players, thereby causing variable degrees of abnormal motility that eventually lead to intestinal neuromuscular disorders. Based on the affected cell type, these disorders can be divided into three subtypes: neuropathies (neuronal defects) that seem to hold true for *CHD8/chd8*, *Slc6a4* and *Nlgn3* [7, 12, 13, 14], myopathies (smooth muscle cell defects), or mesenchymopathies (ICC defects as seen for *Nos1* [8, 9]) (Table 1). However, the development and function of these cells are interconnected, and we need to determine whether a defective cell type is the underlying cause or the consequence of a disorder [16]. To which extent other ASD genes are involved in developmental processes relevant to GI complaints remains to be determined.

**Table 1:** GI manifestations and distress in ASD patients and animal models.

Gene	GI manifestation	GI distress	Study type	ENS phenotype	Reference
*CHD8/chd8*	Slow GI transit	Constipation and diarrhea	ASD patients, *Danio rerio*	50% reduction of enteric neurons in hindgut of *chd8* morphants	Bernier et al. [7]
*NOS1*	Achalasia	Eating and drinking problems	ASD patients	Not analyzed	Shteyer et al. [8]
*Nos1*	Hypertensive and poorly relaxing lower esophageal sphincter	Eating and drinking problems	*Nos* ^−/−^ mice	Interstitial cells of Cajal impaired	Sivarao et al. [9]
*Tcf4*	Slow GI transit	Constipation	*Tcf4* ^+/−^ mice Pitt–Hopkins mouse model	Not analyzed	Grubisic et al. [10]
*Slc6a4 (Sert)*	Slow GI transit, villus height and crypt depth decreased in mucosal structure	Constipation	*Sert Ala56* mice	Reduced number of neurons in plexus of small and large intestines and TH, CGRP, or GABA positive neurons	Margolis et al. [12]
*Foxp1*	Achalasia, impaired relaxation of the lower esophageal sphincter, slow GI transit, reduced thickness of *tunica muscularis* in esophagus and colon	Eating and drinking problems, constipation	*Foxp1* ^+/−^ mice	Not analyzed	Fröhlich et al. [11]
*Nlgn3*	Increased GI transit GABA-mediated alterations of colon motility	Diarrhea	NL3^R451C^ mice	Hyperplasia in small intestine, dysregulation of GABAergic neurons	Hosie et al. [13]
*NLGN3*	Various GI complaints	Diarrhea poor bowel control, esophagitis, esophageal regurgitation, abdominal pain	ASD patients with *NLGN3* p.R451C variant	Not analyzed	Hosie et al. [13]
*Nlgn3*	Faster colonic migrating motor complexes, increased colonic diameter	Not described, likely diarrhea	*Nlgn3* ^−/−^ mice	No changes in ENS, inhibitory interneurons	Leembruggen et al. [14]
*shank3a/shank3b*	Slow GI transit, reduced peristalsis and motility, serotonin-positive enteroendocrine cells reduced	Constipation and/or diarrhea, reflux, cyclical vomiting	*Danio rerio* Phelan-McDermid syndrome (ASD) *shank3abΔC*^*+/*−^	No changes	James et al. [15]

In conclusion, clinical GI symptoms in ASD patients may not just be comorbidities or consequences of medications, but have to be considered as part of the phenotype, just like behavioral symptoms. We therefore propose that these indications should be reflected in patient treatment to improve their GI symptoms and prevent serious sequelae. This may also have a positive impact on the overall well-being of children and adults with ASD, as GI problems are known to exacerbate existing behavioral abnormalities through increasing pain, stress, or discomfort.
